# Infarctus splénique au cours d'une forme grave de la granulomatose avec polyangéite: à propos d'un cas

**DOI:** 10.11604/pamj.2015.21.68.7039

**Published:** 2015-05-28

**Authors:** Olfa Berriche, Wafa Alaya

**Affiliations:** 1 Service de Médecine Interne, Hopital Taher Sfar, Mahdia, Tunisie

**Keywords:** Granulomatose, vascularite, infarctus splénique, granulomatosis, vasculitis, splenic infarction

## Image en medicine

La granulomatose avec polyangéite (GPA) est une vascularite nécrosante systémique, caractérisée par une inflammation granulomateuse, une nécrose tissulaire et une vascularite touchant les vaisseaux de moyen et, surtout, de petit calibre. L'infarctus splénique est une complicationtrès rare de la GPA, sa survenue est grave en raison des potentielles complications hémorragiques pouvant engager le pronostic vital. Nous rapportons l'observation d'un jeune patient âgé de 26 ans, dont le diagnostic de GPA a été posé il y a une semaine, et retenu devant la présence de 3 critères de l'ACR: atteinte ORL, anomalies à la radiologie pulmonaire, et atteinte rénale. Il a présenté subitement des douleurs abdominales prédominant à gauche, l'examen physique a objectivé une sensibilité du flanc gauche. Devant l'aggravation de la symptomatologie digestive, un angioscanner abdominal a été pratiqué montrant un infarctus splénique et un défaut de rehaussement segmentaire du rein droit. Le patient était mis sous corticothérapie et cyclophosphamide. Deux mois plus tard, le patient était ré-hospitalisé pour apparition d'un purpura pétéchial rapidement extensif, une fièvre en rapport avec un sepsis, une aggravation rapide de l'atteinte pulmonaire et rénale à l'origine de son décès.

**Figure 1:**
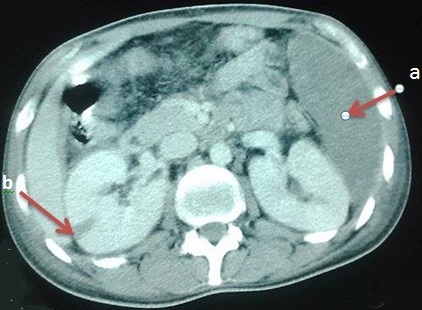
Angio TDM abdominale en coupes axiales: (a) infarctus splénique; (b) un défaut de rehaussement triangulaire du rein droit

